# Percutaneous Vacuum-Assisted Debulking of Infected and Non-Infected Left-Sided Cardiac Masses Using the AngioVac System: A Systematic Review of Published Case Reports and Case Series

**DOI:** 10.3390/jcm15176669

**Published:** 2026-08-28

**Authors:** Felix Bratosin, Jorgelina DeSanctis, Gordana Simeunovic

**Affiliations:** 1 Department of Infectious Disease, Corewell Health West, Grand Rapids, MI 49503, USA; jorgelina.desanctis@corewellhealth.org (J.D.); gordana.simeunovic@corewellhealth.org (G.S.); 2 College of Human Medicine, Michigan State University, Grand Rapids, MI 49503, USA

**Keywords:** AngioVac system, infective endocarditis, vegetation removal, percutaneous intervention, cardiac surgery, systematic review

## Abstract

**Background and Objectives:** Left-sided intracardiac masses (infected vegetations and non-infected thrombotic/tumor lesions) are traditionally managed with surgery in many cases, but procedural risk can be prohibitive. We systematically reviewed published experience with the AngioVac system for percutaneous debulking of left-sided cardiac masses. **Methods:** PubMed, Scopus, and Web of Science were searched for reports through November 2025, supplemented by hand-searching of congress abstract supplements; records were screened and assessed independently by two reviewers. Adult patients undergoing AngioVac removal of infected or non-infected left-sided cardiac masses were included. The primary effectiveness endpoint was technical success (≥70% debulking without conversion to open surgery). Safety outcomes included procedure-related complications. Data were synthesized descriptively due to heterogeneous designs and reporting. Patient-level and study-level data were distinguished a priori, pooled means were weighted by the number of patients contributed by each report, and pre-specified sensitivity analyses addressed possible patient overlap between reports from the same institution and exclusion of a non-intracardiac (aortic arch) target. **Results:** A total of 30 studies were included (predominantly single-patient reports, 90.0%). The pooled cohort comprised 42 patients; 23/42 (54.8%) had infective endocarditis (IE) and 45.2% had non-infected masses. Mean age was 62.9 years (range 30.0–86.0); sex distribution was female 47.6%, male 42.9%, and not reported 9.5%. All 42 patients were deemed to be at a prohibitively high risk of surgery. Access was mainly transseptal (29/42, 69.0%) or transapical (8/42, 19.0%). Mass size was reported in 23/42 (54.8%), with mean 23.3 mm, median 20.0 mm, and range 11.0–57.0 mm. Technical success was achieved in 35/42 (83.3%); residual mass occurred in 6/42 (14.3%) and one patient had cardiac perforation requiring conversion to open heart surgery (1/42, 2.4%). Procedure-related complications included valvular dysfunction (paravalvular leak or progressive mitral regurgitation), cardiac perforation, and cerebral infarctions (4/42, 9.5%). There were two deaths (2/42, 4.8%) after successful debulking, one in-hospital with recurrent embolic events and septic shock, and the other 4 months after the procedure following procedure-related progressive mitral regurgitation. Complications clustered in transapical procedures (3/8, 37.5% vs. 0/29 transseptal; *p* = 0.007) and in fungal endocarditis (2/2; *p* = 0.012). New clinically apparent embolic events occurred in 2/42 (4.8%), both in patients without documented cerebral protection, the choice of which to apply in the total population was unrelated to mass size (mean 23.5 vs. 23.2 mm). Estimates were stable in sensitivity analyses (technical success 32/38, 84.2% after maximal-overlap de-duplication; 31/37, 83.8% restricted to strictly intracardiac targets). Length of stay was reported in 8/42 (19.0%) with a median of 3.5 days (range 1.0–30.0). **Conclusions:** In published, highly selected non-surgical candidates, left-sided AngioVac debulking achieved ≥70% mass reduction in 83% of the 42 patients, with 4.8% mortality and one patient (2.4%) converting to emergency surgery. Because the evidence consists almost entirely of single-patient reports subject to publication bias, and because technical success is operator-reported and did not preclude death, these data do not support substituting debulking for a guideline-indicated operation. The technique is best positioned as a bridge or palliative strategy, with transapical access and fungal aetiology identified as signals warranting particular caution.

## 1. Introduction

Left-sided cardiac masses, including infective vegetations, thrombi, and cardiac tumours are associated with significant morbidity and mortality due to their potential to cause systemic embolization, valvular dysfunction, heart failure, and hemodynamic compromise [1,2]. Conventional management frequently involves open surgical interventions for source control, embolic risk reduction, or restoration of valve function. However, a significant proportion of patients are considered poor surgical candidates because of advanced age, multiple comorbidities, hemodynamic instability, prior cardiac surgery, or prohibitive operative risk [3,4,5].

The clinical burden that motivates these interventions is substantial. In the prospective ESC-EORP EURO-ENDO registry of 3116 patients, in-hospital mortality was 17.1% and embolic events occurred in 20.6%; cardiac surgery was theoretically indicated in 69.3% of patients but was ultimately performed in only 73.9% of those in whom it was indicated, and failure to undertake surgery when indicated was an independent predictor of death, as was vegetation length greater than 10 mm [6]. A meta-analysis of 21 studies comprising 6646 patients confirms that a vegetation exceeding 10 mm carries more than double the odds of embolism (odds ratio 2.28, 95% CI 1.71–3.05) and increased odds of death (odds ratio 1.63, 95% CI 1.13–2.35) [7], with the embolic hazard concentrated before and during the earliest days of antimicrobial therapy [8]. Most directly relevant to the present review is the outcome of patients who have a formal surgical indication but are not operated upon, since this is precisely the population in which percutaneous debulking is considered. In a consecutive series of 1105 patients with left-sided infective endocarditis, 33% of those with a formal surgical indication received medical therapy alone; their in-hospital and one-year mortality were 46% and 57.1% respectively, compared with 24% and 27.6% in operated patients. High surgical risk was the stated reason for rejection in 57.1% of cases, and in-hospital mortality was 45% when the surgical indication was prevention of embolism, which is the indication most often invoked for percutaneous debulking [9]. Mortality is similarly high in left-sided endocarditis complicated by heart failure [5], and non-infected left-sided masses carry an analogous burden, with obstructive prosthetic valve thrombosis and anticoagulation-refractory left atrial thrombus both conferring substantial mortality and stroke risk [1,10]. These figures define both the unmet need that percutaneous debulking seeks to address and the outcome benchmark against which any such strategy must ultimately be judged.

Over the past decade, minimally invasive and catheter-based approaches have emerged as alternatives for selected high-risk patients with intracardiac masses [11,12]. Among these technologies, the AngioVac system (AngioDynamics, Inc., Latham, NY, USA) is a percutaneous vacuum-assisted aspiration device initially designed for the removal of venous thrombi and right-sided cardiac masses [13,14,15]. The system enables extracorporeal aspiration, filtration, and reinfusion of blood while debulking intravascular material. Although its use has been most extensively described in right-sided infective endocarditis and venous thromboembolic disease, growing procedural experience has expanded its application to selected left-sided cardiac lesions [16,17,18,19].

Percutaneous debulking of left-sided cardiac masses presents unique clinical and technical challenges compared with right-sided interventions, including the risk of systemic embolization, neurological injury, valvular damage, and hemodynamic deterioration [17,18]. Despite these concerns, published experience suggests that, in carefully selected patients who are not surgical candidates, left-sided AngioVac debulking may provide a feasible alternative or bridge strategy for reducing mass burden and embolic risk, although comparative data remain lacking [19,20,21,22].

Current evidence regarding left-sided AngioVac use remains limited and consists predominantly of case reports and small case series with heterogeneous patient populations, procedural approaches, and outcome reporting. Furthermore, infected vegetations and non-infected masses represent biologically distinct entities with different therapeutic objectives and risk profiles. As a result, the overall effectiveness, procedural safety, and clinical role of left-sided AngioVac debulking remain incompletely defined.

This systematic review aims to synthesize the available literature on percutaneous vacuum-assisted debulking of infected and non-infected left cardiac masses using the AngioVac system. We examined patient characteristics, procedural approaches, technical success, and reported complications in order to better characterize the currently available evidence and identify areas requiring further investigation.

## 2. Materials and Methods

### 2.1. Eligibility Criteria and Information Sources

This systematic review followed the Preferred Reporting Items for Systematic Reviews and Meta-Analyses (PRISMA) guidelines (Table S1). The study protocol was registered with the Open Science Framework (registration code 10.17605/OSF.IO/N384U) [23]. We included eligible studies involving adult patients diagnosed with infected or non-infected left-sided cardiac masses who underwent mass removal using the AngioVac system. Our review encompassed both prospective and retrospective studies, as well as case reports, and case series published through November 2025. Abstracts presented at scientific congresses were also eligible for inclusion, provided that they reported sufficient patient-level data on the intervention and its outcome. Exclusion criteria included studies focused exclusively on right-sided cardiac masses, review articles, editorials, and non-English publications. Data sources included PubMed, Scopus, and Web of Science databases.

### 2.2. Search Strategy

A comprehensive search strategy incorporated relevant keywords and Medical Subject Headings (MeSH) terms related to the AngioVac system and left-sided cardiac masses. The selected search terms included “AngioVac,” “mitral valve,” “aortic valve,” “left heart masses,” “intracardiac thrombus,” “cardiac tumor,” “cardiac vegetation,” “percutaneous vegetectomy,” and “minimally invasive cardiac surgery.” Boolean operators were used to create database-specific search strings tailored for each major database:

PubMed: ((“AngioVac”[Title/Abstract]) AND (“mitral valve”[Title/Abstract] OR “aortic valve”[Title/Abstract]) AND (“mass”[Title/Abstract] OR “thrombus”[Title/Abstract] OR “tumor”[Title/Abstract] OR “vegetation”[Title/Abstract])) OR (“minimally invasive cardiac surgery”[MeSH Terms] AND “percutaneous vegetectomy”[Title/Abstract]).

Scopus: (TITLE-ABS-KEY(“AngioVac”) AND (TITLE-ABS-KEY(“mitral valve”) OR TITLE-ABS-KEY(“aortic valve”))) AND (TITLE-ABS-KEY(“left heart masses”) OR TITLE-ABS-KEY(“intracardiac thrombus”) OR TITLE-ABS-KEY(“cardiac tumor”) OR TITLE-ABS-KEY(“cardiac vegetation”)) AND TITLE-ABS-KEY(“percutaneous vegetectomy”).

Web of Science: (TS = (“AngioVac”) AND (TS = (“mitral valve” OR “aortic valve”))) AND (TS = (“left heart masses” OR “intracardiac thrombus” OR “cardiac tumor” OR “cardiac vegetation”)) AND TS = (“percutaneous vegetectomy”).

### 2.3. Study Selection and Data Extraction

Two reviewers (F.B., first reviewer, and G.S., second reviewer) independently screened titles and abstracts and assessed full texts for eligibility against the predefined criteria, with disagreements resolved by consensus. The full texts of all included reports were read by both reviewers, and the second reviewer (G.S.) additionally repeated the database searches and hand-searched congress abstract supplements to verify the completeness of study retrieval. Studies that involved patients with infected or non-infected left-sided cardiac masses treated using the AngioVac system were included. Full-text articles identified as potentially relevant were retrieved for a detailed assessment to confirm their eligibility.

Because an evidence base dominated by single-patient reports arising from a small number of high-volume structural heart programmes carries a material risk that the same patient is published more than once, a formal de-duplication procedure was applied before analysis. All included reports were cross-tabulated by author names, institutional affiliation, city, recruitment period and patient-level descriptors (age, sex, infective versus non-infective aetiology, causative organism, target anatomy, access route, mass size and outcome). Two clusters of reports were identified as carrying a material risk of overlap, each comprising several single-patient reports and one institutional series from the same centre, with substantially overlapping authorship and an accrual window that could encompass the individually reported patients; these clusters are identified in Section 3.2. Because no report provided patient identifiers, admission dates or descriptors granular enough to confirm or exclude overlap definitively, all patients were retained in the primary analysis, that is, the maximum-count scenario, and a pre-specified maximal-overlap sensitivity analysis was performed in which every single-patient report that could plausibly be subsumed within a same-institution series was removed (Section 3.6). Reports from different institutions, or describing patients with discordant age, sex, aetiology or target anatomy, were considered to represent distinct patients. In addition, because one patient in the largest series underwent extraction of a mass located in the aortic arch, an extracardiac intravascular target lying outside the definition of a left-sided intracardiac mass, a second pre-specified sensitivity analysis was restricted to strictly intracardiac targets.

### 2.4. Quality Assessment and Risk of Bias

Formal quality assessment was limited by the predominance of case reports [24,25]. Because the Newcastle–Ottawa Scale is not applicable to single-patient reports [24], methodological quality was appraised using the Joanna Briggs Institute critical appraisal checklists for case reports and for case series [25], assessing clarity of patient and lesion description, completeness of the procedural account, adequacy of outcome ascertainment and reporting of adverse events. Across the 30 included reports the recurrent deficiencies were the absence of a pre-specified or objectively verified definition of procedural success, incomplete reporting of follow-up duration and of the criteria used to declare a patient inoperable, and selective reporting of favourable outcomes, with the four conference abstracts additionally limited by the absence of a peer-reviewed full text. Because the resulting appraisal was uniformly of low certainty and did not discriminate between reports, it was used to inform interpretation rather than to weight or exclude studies. Risk of bias at the review level, principally publication bias favouring successful or novel procedures was also addressed.

### 2.5. Outcome Measures and Data Synthesis

Because standardized endpoints are lacking in this field, technical success was pragmatically defined as removal of ≥70% of the left-sided cardiac mass using the AngioVac system without conversion to open surgery, as reported by the treating team and/or supported by post-procedure imaging. The primary safety outcome was the occurrence of procedure-related complications, including (but not limited to) systemic embolic events, neurologic injury, valvular damage or dysfunction (new or worsening regurgitation or paravalvular leak), cardiac or vascular perforation, hemodynamic deterioration, bleeding or coagulopathy, need for unplanned reintervention, and death. Secondary outcomes included patient demographics (age and sex), clinical indication (infective endocarditis versus non-infected mass), microbiologic findings among infective endocarditis cases, procedural characteristics (access route, target anatomy, use and type of cerebral embolic protection, and mass size when explicitly reported), and post-procedure course metrics (length of hospitalization and duration of follow-up when reported).

Because eligible reports were heterogeneous in design and outcome definitions and frequently provided incomplete or non-uniform reporting, data were synthesized using qualitative methods supported by descriptive statistics. Categorical variables were summarized as counts and proportions. Continuous variables were summarized as means and ranges when individual patient values were provided and as study-reported means when only aggregate values were available; no imputation was performed. Formal meta-analysis was not conducted due to the predominance of case reports and small case series, small sample sizes, and inconsistent reporting of variance measures and follow-up intervals.

Because the included reports mixed individual patient descriptions with study-level summary statistics, the level at which each variable was analysed was defined a priori and is stated explicitly throughout the Results. Sex, aetiology, causative organism, access route, target anatomy, use of cerebral embolic protection, technical success, complications and mortality were available at the individual patient level for all 42 patients and are reported as patient-level counts and proportions with 42 as the denominator. Age was available at the individual patient level for 28 patients, was not reported in one congress abstract, and only as a study-level mean for the two largest series (n = 10, mean 58.3 ± 17.3 years; and n = 3, mean 58.5 ± 16.5 years). The pooled mean age was therefore calculated as a sample-size-weighted average, in which each report contributed its mean multiplied by the number of patients it described and the sum of these products was divided by the total number of patients (Σ[nᵢ × meanᵢ]/Σnᵢ = 2577.5/41 = 62.9 years); the same weighting was applied to any other continuous variable reported only as a study-level mean. Reported minima and maxima, for example the age range of 30.0–86.0 years, are derived exclusively from individual patient values and therefore exclude patients described only within study-level means, and medians and ranges for mass size were likewise computed from individual values only. Because standard deviations were unavailable for most reports, no pooled measure of dispersion was calculated and no inverse-variance pooling was undertaken; the weighting described here is a sample-size weighting only and does not constitute a meta-analysis.

One report provided technical success only as a study-level proportion, namely 80% of 10 patients. This was converted to patient-level counts by applying the reported proportion to the reported denominator, yielding eight successes and two patients with residual mass; because the report did not identify which individual patients accounted for the 20% non-success proportion, these two patients could not be characterised further and contributed to the numerator of residual mass but not to any patient-level subgroup analysis.

### 2.6. Assessment and Adjudication of Technical Success

Because no validated endpoint exists for percutaneous left-sided mass debulking, technical success required documentation of removal of ≥70% of the target mass together with the absence of conversion to open surgery during the index hospitalisation. The 70% threshold was adopted pragmatically because it is the degree of debulking most commonly cited in the AngioVac literature as sufficient to reduce embolic burden and because it is discriminable by the semi-quantitative imaging used in practice; it has not been prospectively validated against clinical outcomes, and this is acknowledged as a limitation.

The modality used to assess residual mass differed across reports, and no attempt was made to re-measure lesions from published images. A three-step harmonisation procedure was therefore applied. First, the modality and timing of the residual-mass assessment were extracted verbatim for every report. Second, each report was assigned to one of three pre-specified evidence tiers: Tier 1, quantitative comparison of pre- and post-procedural mass dimensions on transoesophageal echocardiography or computed tomography; Tier 2, qualitative post-procedural imaging described as showing complete or near-complete resolution without stated measurements; and Tier 3, operator visual estimate of aspirated material or an unqualified narrative statement of success without corroborating post-procedural imaging. Third, each report was adjudicated against the ≥70% threshold using the highest tier of evidence available, with reports whose highest available evidence was Tier 3 classified as successful only where the narrative explicitly described complete or near-complete removal; ambiguous descriptions were classified as not meeting the threshold, and any report describing residual mass without quantification was classified as residual mass rather than technical success. This approach is deliberately conservative and will tend to underestimate success. Adjudication was performed independently by both reviewers (F.B. and G.S.) using the tier definitions above, with disagreements resolved by consensus.

### 2.7. Pre-Specified Subgroup and Risk-Factor Analyses

Exploratory analyses examined the association between pre-specified candidate risk factors and the occurrence of clinically significant procedure-related complications, defined as any of cardiac perforation, new or worsening valvular dysfunction, new clinically apparent embolic or cerebrovascular event, conversion to emergency surgery or death. Candidate factors were access route (transseptal versus transapical versus retrograde arterial), target anatomy (native valve versus prosthetic material versus non-valvular), mass size dichotomised at 20 mm (the median of reported values), causative organism category (staphylococcal versus streptococcal or enterococcal versus fungal versus culture-negative or not reported), and use of cerebral embolic protection. The relationship between cerebral embolic protection and mass size was examined separately, both as a comparison of means and as a comparison of the proportion of masses ≥ 20 mm.

Between-group comparisons used Fisher’s exact test for categorical variables, given the small expected cell counts, and are reported as two-sided *p* values; continuous variables are compared descriptively. These analyses are exploratory and hypothesis-generating: they aggregate case-level data drawn from reports with heterogeneous ascertainment, they are not adjusted for multiple comparisons or for confounding by indication, and they are powered only to detect very large effects. They are presented to identify signals for prospective evaluation and must not be interpreted as establishing causal risk factors. Statistical computations were performed in Python 3.11 using the SciPy library (version 1.11.4).

## 3. Results

### 3.1. Study Selection

**Figure 1:**
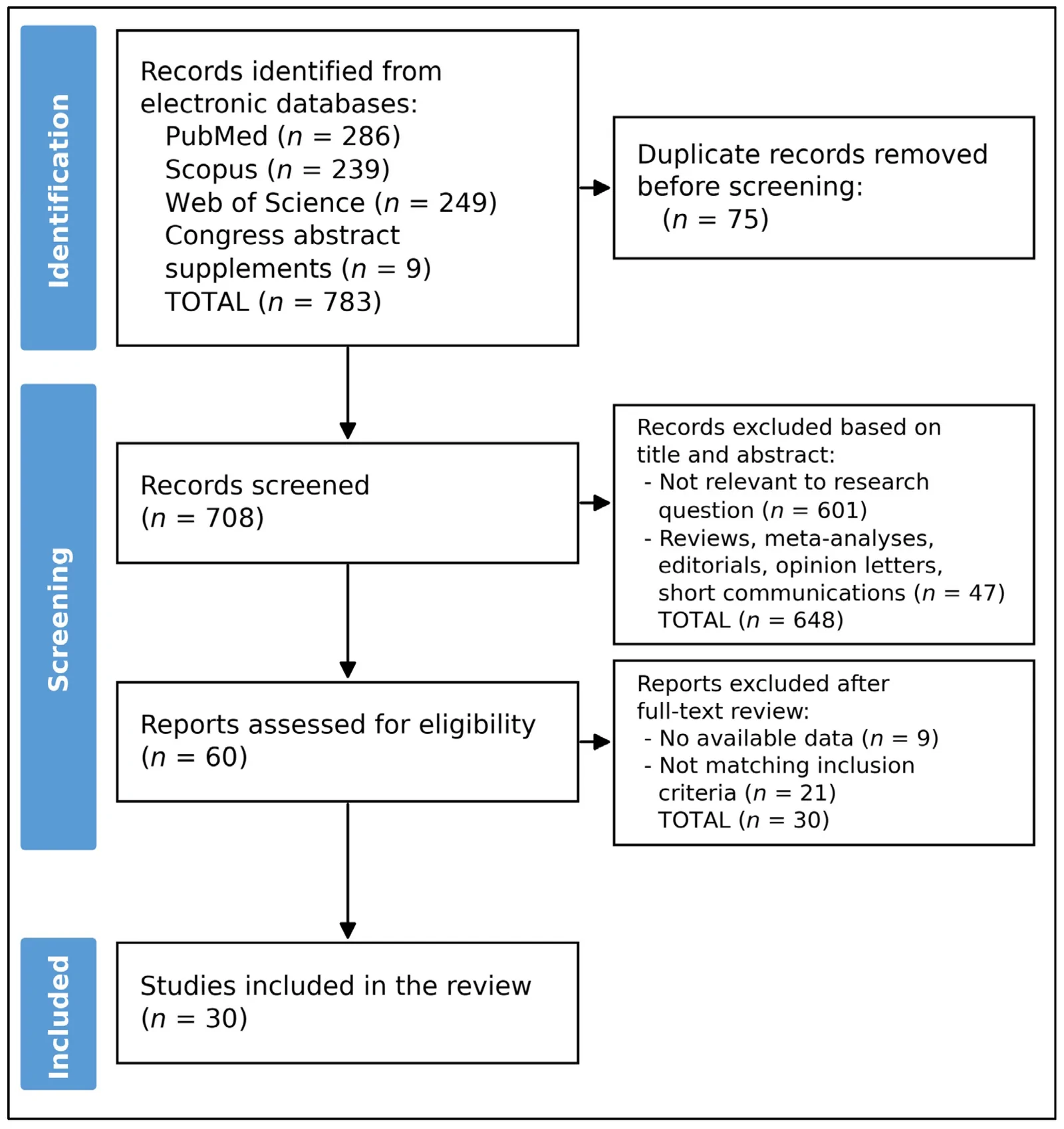
PRISMA flowchart diagram.

The literature search identified 774 records across electronic databases (PubMed, n = 286; Scopus, n = 239; Web of Science, n = 249), and hand-searching of congress abstract supplements identified nine additional records. Following title and abstract review, 75 duplicate records were first removed, leaving 708 records for title and abstract screening. Of these, 648 records were excluded, primarily because they were not relevant to the research question (n = 601) or because they were not eligible publication types (reviews, meta-analyses, editorials, opinion letters, or short communications; n = 47). This left 60 reports that were assessed for eligibility. Following full-text review, 30 reports were excluded due to unavailable data (n = 9) or failure to meet the inclusion criteria (n = 21). In total, 30 studies were included in the final review (Figure 1).

### 3.2. Study Characteristics

Across 30 included reports, the evidence base was dominated by single-patient reports (90.0%), comprising 23 case reports (76.7%) and four congress abstracts (13.3%), with only three small case series (10.0%). Publication years spanned 2019–2025, and most reports originated from the USA (66.7%), followed by Italy (13.3%). Country of origin was not reported in 10.0%, while Switzerland, Canada, and Qatar each contributed one study (3.3%), as presented in Table 1.

Cross-tabulation of authorship and institutional affiliation identified two clusters of reports at material risk of describing overlapping patients. Four reports (13.3%) originated from a single Detroit structural heart programme [27,32,36,38] and three (10.0%) from a single Padua programme [30,33,44]; within each cluster, one report is an institutional series [32,44] whose case accrual could encompass the individually reported patients. Because no report provided identifiers or admission dates permitting definitive adjudication, all patients were retained in the primary analysis, and the maximal-overlap and strictly intracardiac sensitivity analyses presented in Section 3.6 quantify the effect of this uncertainty on every principal estimate. Author overlap was also noted between two single-patient congress abstracts from the same group [39,53] and among single-patient reports from the same tertiary centre [41,45,54]; in each instance, discordant patient age, sex or clinical descriptors excluded duplication, and these reports were therefore treated as non-duplicative.

### 3.3. Patient Characteristics

**Figure 2:**
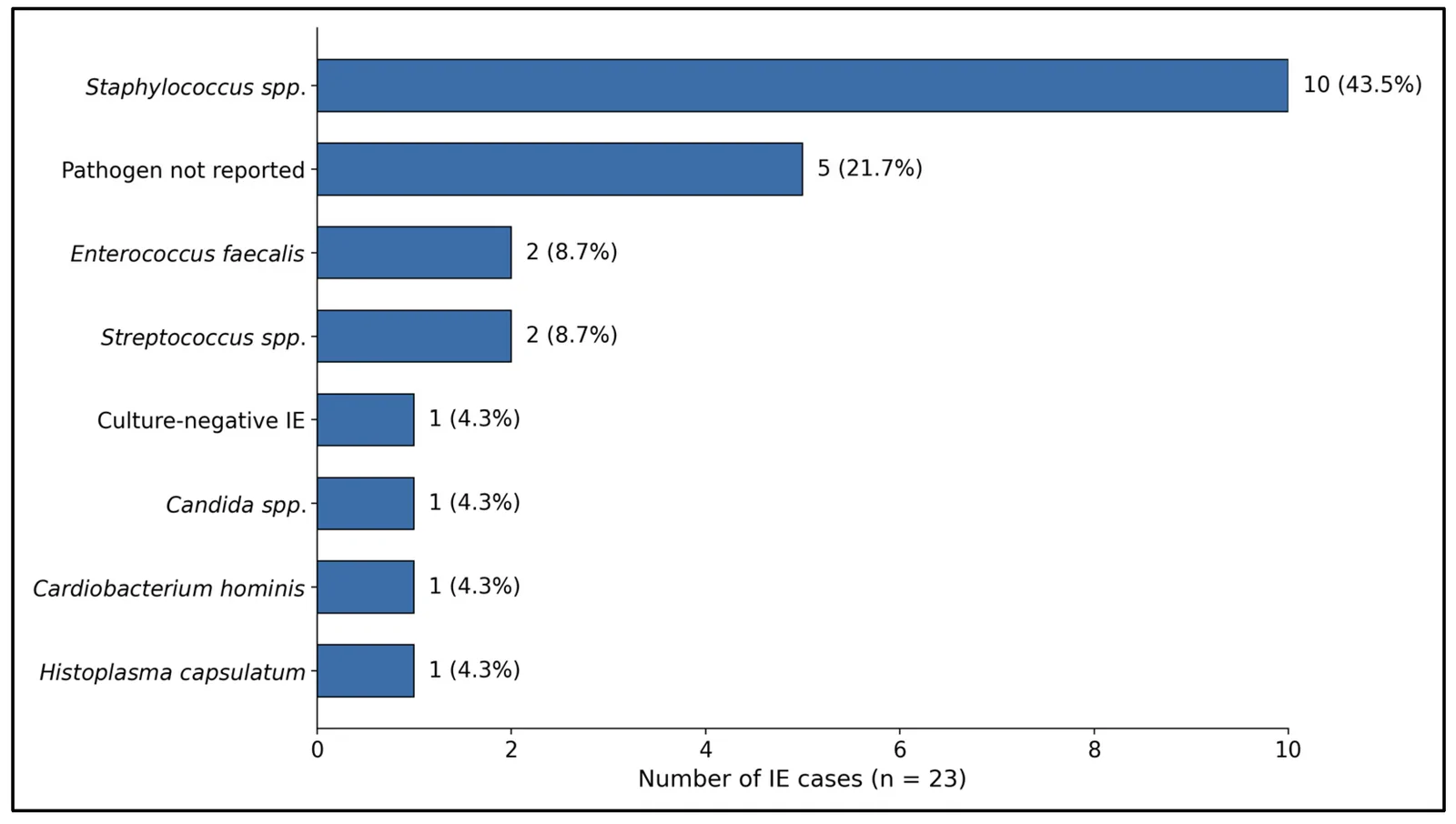
Microbiologic spectrum in infective endocarditis cases.

The pooled cohort comprised 42 patients, with IE present in 23/42 (54.8%) and non-infected masses in 19/42 (45.2%). Of the two patients reported by Fiocco et al. [28], only one had infective endocarditis; the second underwent debulking of a non-infected lesion, and this patient-level split is reflected in the totals above. Sex distribution was balanced (female 47.6%, male 42.9%, NR 9.5%), while the age distribution skewed older overall (mean age 62.9 years; range 30.0–86.0 years). Among IE cases (n = 23), the most frequently reported organisms were *Staphylococcus* spp. (10/23, 43.5%; including MSSA/MRSA and other staphylococci), while *Enterococcus* spp. accounted for 2/23 (8.7%), as described in Table 2 and Figure 2.

Intracardiac prosthetic material was present in a substantial minority of patients. A prosthetic valve was the target of, or immediately adjacent to, the debulked mass in 10/42 patients (23.8%): a mitral bioprosthesis or mechanical mitral prosthesis in eight patients [30,34,42,46,49,50,51,52], combined mitral and aortic prostheses in one patient [28], and a bioprosthetic aortic valve in one patient [48]. One further 2.4% of patients had thrombus adherent to a left atrial appendage occluder device [27]; therefore, intracardiac prosthetic material of any type was present in 11/42 patients (26.2%). Prosthetic valve involvement was more frequent among patients with infective endocarditis (7/23, 30.4%) than among those with non-infected masses (3/19, 15.8%).

Cardiac implantable electronic devices were not systematically reported. No included report described a lead-associated vegetation as the debulking target or concomitant device or lead extraction at the time of the index procedure, and explicit statements confirming the presence or absence of a pacemaker or defibrillator were absent from the great majority of reports. The number of patients carrying such a device therefore could not be determined and is deliberately not presented as a proportion. This is a specific and consequential reporting gap since device carriage is both a recognised substrate for infective endocarditis and a determinant of whether percutaneous debulking can achieve source control.

All included reports described the intervention as having been undertaken in patients considered to be at prohibitive or very high surgical risk, but the basis for that judgement was documented in narrative form only. No report provided a calculated operative risk score such as EuroSCORE II or the STS Predicted Risk of Mortality, and no report described the composition or formal deliberation of a multidisciplinary heart team. The reasons cited fell into six recurring categories: cumulative comorbidity burden with frailty and advanced age; prior cardiac surgery or previous sternotomy conferring redo-operative risk; haemodynamic instability, cardiogenic or septic shock, or ventilator dependence at the time of intervention; recent cerebral infarction or intracranial haemorrhage rendering immediate cardiopulmonary bypass hazardous; ongoing sepsis or uncontrolled infection judged to confer unacceptable perioperative risk; and patient or family refusal of surgery. Several reports cited more than one reason, and a minority framed the procedure explicitly as a bridge to later surgery rather than as definitive therapy. Because these justifications were unstandardised, frequently brief and not mutually exclusive, they could not be reliably enumerated as patient-level proportions, and the comparability of this population with surgically treated cohorts therefore cannot be established.

### 3.4. Procedural Characteristics

**Figure 3:**
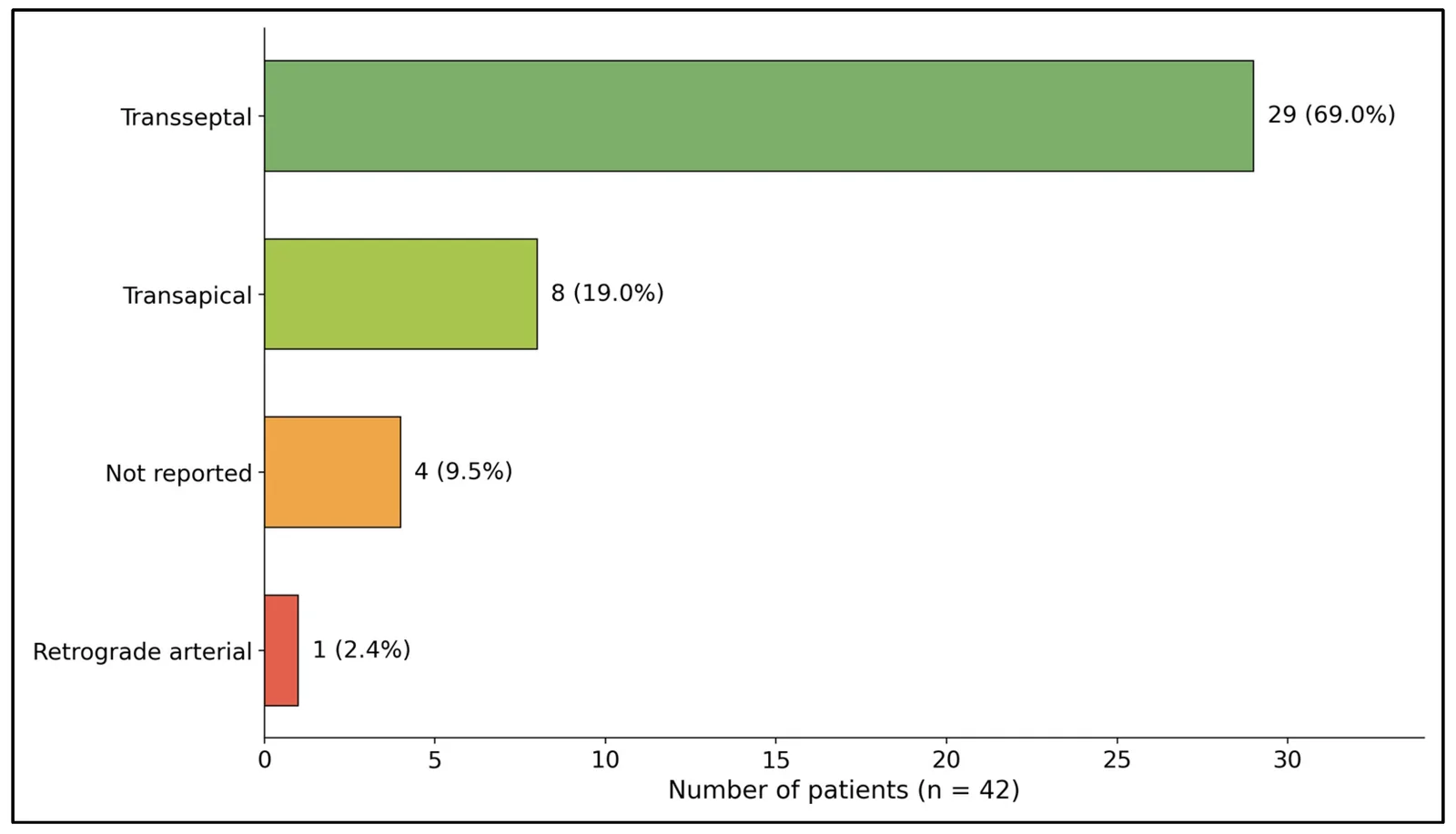
Access routes for left-sided AngioVac debulking.

Debulking was performed predominantly via transseptal access (29/42, 69.0%), with transapical approaches used in 8/42 (19.0%), retrograde arterial access in 1/42 (2.4%), and access not reported in 4/42 (9.5%) (Figure 3). Target anatomy predominantly involved the mitral valve: mitral-only locations comprised 25/42 (59.5%), while mitral + aortic comprised 12/42 (28.6%); isolated aortic-only and LAA-only targets were each 2/42 (4.8%), and the target location was not reported in 1/42 (2.4%). Embolic/cerebral protection reporting was variable: SENTINEL™ (Boston Scientific, Marlborough, MA, USA) was documented in 20/42 (47.6%), other or unspecified devices in 6/42 (14.3%), and not reported in 16/42 (38.1%). Mass size was reported for 23/42 (54.8%), with a mean of 23.3 mm, median 20.0 mm, and range 11.0–57.0 mm (Table 3).

The relationship between cerebral embolic protection and mass burden was examined directly. Among the 23 patients for whom mass size was reported, mean mass size was 23.5 mm (median 20.0 mm; range 11.0–55.0) in the 11 patients in whom a protection device was documented and 23.2 mm (median 20.0 mm; range 12.0–57.0) in the 12 patients in whom no device was reported. The proportion of masses ≥ 20 mm was likewise similar (7/11, 63.6% with protection versus 7/12, 58.3% without; *p* = 1.00, Fisher’s exact test). There was no observed association between mass size and use of cerebral protection; of the two largest masses in the cohort, one (55 mm) was treated with a TriGuard device (Keystone Heart, Caesarea, Israel) [50] and the other (57 mm) without documented protection [46]. Device use instead tracked with institution and publication year, since every report documenting protection appeared in 2020 or later and the SENTINEL™ device accounted for 20 of 26 protected patients (76.9%).

### 3.5. Procedural Outcomes and Safety

Using the effectiveness definition (≥70% debulking), technical success was reported in 35/42 (83.3%), while residual mass was documented in 6/42 (14.3%), including the 20% non-success proportion reported in the largest cohort [32]. One further patient (Tarzia et al. [44]) sustained cardiac perforation that required conversion to emergent open heart surgery and was therefore not classified as a technical success (1/42, 2.4%). Reported technical success should be interpreted cautiously because objective assessment of residual mass was inconsistently reported and success definitions varied considerably across studies. Two deaths were reported (2/42, 4.8%). One patient experienced recurrent embolic events followed by septic shock and death [48], while another patient developed progressive mitral regurgitation and died 4 months after the procedure [40]. Clinically significant procedure-related complications occurred in four patients (4/42, 9.5%) and comprised paravalvular leak [28], progressive mitral regurgitation, which began early after the procedure as a procedure-related valvular complication and progressed until the patient’s death at 4 months [40], cardiac perforation [44], and multiple cerebral infarctions [52]. Length of stay was inconsistently reported (eight patients), ranging 1.0–30.0 days (median 3.5). Follow-up duration, when available, ranged from several weeks to approximately 30 months (Table 4).

Applying the evidence-tier scheme described in Section 2.6, quantitative pre- and post-procedural measurement (Tier 1) was available for 7 of 30 reports (23.3%), qualitative post-procedural imaging (Tier 2) for 16 (53.3%) and operator narrative alone (Tier 3) for 7 (23.3%); 23 of 30 reports (76.7%) therefore provided only Tier 2 or Tier 3 evidence, and the pooled success estimate rests predominantly on operator-reported assessment.

Outcomes were also examined according to their timing. Intraprocedural complications were reported in 1/42 patients (2.4%), namely the cardiac perforation described by Tarzia et al. [44], which required immediate conversion to open heart surgery and which the patient survived. No intraprocedural death occurred, and no report described massive transfusion, circuit-related haemolysis, air embolism, device entrapment or access-site vascular injury; whether such events were genuinely absent or simply unreported cannot be determined, since no report used a standardised intraprocedural complication checklist. In-hospital or 30-day complications were reported in 2/42 patients (4.8%), comprising that perforation and the recurrent embolic events with subsequent septic shock reported by Qafisheh et al. [48], and in-hospital or 30-day all-cause mortality was 1/42 (2.4%). One further death occurred beyond 30 days, four months after technically successful transapical debulking of an anterior mitral leaflet vegetation in a patient who developed progressive mitral regurgitation [40], giving a total all-cause mortality of 2/42 (4.8%) across all available follow-ups.

Events judged by the reporting authors to be directly attributable to the procedure comprised the cardiac perforation [44], the paravalvular leak identified at one month [28] and the progressive mitral regurgitation preceding the late death [40], giving a procedure-attributable complication rate of 3/42 (7.1%) and a procedure-attributable mortality of 1/42 (2.4%). The cerebral infarctions reported by Affas et al. [52] and the recurrent embolic events reported by Qafisheh et al. [48] were attributed by their authors to ongoing endocarditis rather than to instrumentation, but both occurred in the periprocedural window and neither patient had documented cerebral embolic protection; counting these as possibly procedure-related raises the upper bound of procedure-attributable complications to 5/42 (11.9%).

Systemic embolism was both an indication for and a complication of the procedure. Embolic phenomena were the presenting manifestation prompting intervention in at least three patients, all of whom presented with embolic ischaemic stroke before debulking [39,52,54], while new clinically apparent embolic events after the procedure occurred in 2/42 patients (4.8%), comprising the multiple cerebral infarctions reported by Affas et al. [52] and the recurrent embolic events preceding septic shock reported by Qafisheh et al. [48]. Both of the latter were treated without documented cerebral embolic protection, whereas none of the 26 patients in whom a protection device was documented sustained a reported new cerebrovascular event (0/26 versus 2/16; P = 0.14, Fisher’s exact test). No report described systematic post-procedural neuroimaging or formal neurological assessment of asymptomatic patients, so subclinical cerebral embolization, the event most consistently demonstrated by filter-capture analyses in the transcatheter aortic valve literature, was not ascertained in any patient and its true incidence is unknown.

Subsequent cardiac surgery was infrequently reported. Conversion to emergency open heart surgery during the index procedure occurred in 1/42 patients (2.4%) [44]. No report described a planned, delayed surgical valve replacement performed after successful debulking, and no report stated that a patient was re-referred for surgical evaluation once clinically stabilised. Two patients (4.8%) underwent subsequent transcatheter rather than surgical intervention, namely a mitral valve-in-valve procedure after vegetation aspiration [34] and percutaneous mitral balloon valvuloplasty after left atrial appendage thrombectomy [38], in both cases with debulking performed to enable the definitive percutaneous procedure. Whether the remaining patients were subsequently offered surgery was not reported, so the frequency with which left-sided debulking functions as a true bridge to surgery, as opposed to definitive or palliative therapy, cannot be determined from the published literature.

### 3.6. Sensitivity Analyses for Patient Overlap and Target Location

Three analysis sets were compared. In the primary, as-reported analysis (n = 42), technical success was 35/42 (83.3%), residual mass 6/42 (14.3%), conversion to emergency surgery 1/42 (2.4%) and all-cause mortality 2/42 (4.8%). In a maximal-overlap analysis, every single-patient report that could plausibly be subsumed within a same-institution series was removed, namely Frisoli et al. [27], Antoun et al. [36] and So et al. [38] as potentially within the Qintar et al. series [32], and Gerosa et al. [30] as potentially within the Tarzia et al. series [44]. This reduced the cohort to 38 patients, among whom technical success was 32/38 (84.2%), residual mass 5/38 (13.2%), conversion 1/38 (2.6%) and mortality 2/38 (5.3%). Gerosa et al. [33] was retained because it describes non-infective prosthetic thrombosis whereas all three patients in the Tarzia series had infective endocarditis, rendering overlap implausible.

Restricting that cohort further to strictly intracardiac targets, by excluding the single patient in the Qintar series [32] whose mass lay in the aortic arch and conservatively assuming that patient to have been a technical success, gave 37 patients with technical success 31/37 (83.8%), residual mass 5/37 (13.5%), conversion 1/37 (2.7%) and mortality 2/37 (5.4%). Technical success therefore varied only between 83.3% and 84.2% and all-cause mortality only between 4.8% and 5.4% across the three sets, indicating that the principal conclusions of this review are robust both to plausible patient duplication and to the inclusion of a single non-intracardiac target.

## 4. Discussion

### 4.1. Assessment of Findings and Additional Literature

The present synthesis suggests that percutaneous vacuum-assisted debulking of left-sided cardiac masses can achieve high immediate technical success in a cohort composed predominantly of high-surgical-risk patients, with technical success (≥70% debulking) reported in 83.3% of cases and residual mass in 14.3%, with one additional patient (2.4%) requiring conversion to emergent open heart surgery for cardiac perforation. These outcomes are notable given the substantial mean reported mass size of 23 mm and frequent mitral involvement, features that, when occurring in infective endocarditis, are traditionally associated with elevated embolic risk and are commonly addressed with early surgical strategies in guideline-driven care pathways [7,56,57]. Importantly, reported technical success should not be equated with favourable clinical outcomes. In the present review, two patients who underwent successful debulking subsequently died, highlighting the need to distinguish technical success from clinical outcomes. Furthermore, the apparent technical success observed in the published literature should be interpreted in the context of limited and heterogeneous follow-up. Most patients lacked standardized long-term outcome data, precluding reliable assessment of durability, recurrence, and the need for subsequent surgical intervention.

Given these uncertainties and the limited long-term outcome data, contemporary guidance continues to emphasize surgery as the standard approach for left-sided IE when there is heart failure, uncontrolled infection, or a strong embolic prevention indication, while randomized evidence, such as early surgery for large vegetations, supports embolic-risk reduction in selected patients [56,57]. Therefore, left-sided AngioVac debulking should be interpreted as an adjunctive or alternative strategy mainly for patients in whom surgery is prohibitive or deferred, rather than a replacement for established surgical indications.

An important clinical observation is that approximately half of the reported patients underwent intervention for IE (54.8%), while a substantial proportion had non-infected masses (45.2%), underscoring that “left-sided mass extraction” encompasses heterogeneous disease biology and therapeutic goals. Among IE cases, the predominance of *Staphylococcus* spp. aligns with modern epidemiology in which healthcare exposure, intracardiac prostheses, and device-related substrates are common, and it reinforces the importance of multidisciplinary decision-making that integrates microbiologic clearance, embolic risk, and valve function [56,58]. Importantly, embolic events in IE cluster early and evolve with antimicrobial therapy; contemporary observational data evaluating embolic events before and after antibiotic initiation further supports the need to consider time-sensitive risk mitigation when contemplating any mechanical debulking strategy [8]. In this context, percutaneous debulking may be most defensible as a bridge (to definitive surgery or to stabilization), or as palliation/embolism-risk reduction when definitive options are not feasible.

From a procedural standpoint, the predominance of transseptal access (69.0%) and frequent mitral targeting highlights that left-sided AngioVac use is fundamentally a structural-heart intervention requiring meticulous echocardiographic guidance and careful embolic planning. Over half of cases documented cerebral embolic protection, reflecting operator concern for systemic embolization during left-heart manipulation. Although direct evidence for embolic protection in left-sided vegetectomy is limited, randomized trials of cerebral embolic protection during transcatheter aortic valve replacement demonstrate consistent debris capture and suggest potential benefit signals for disabling stroke in some analyses, while overall stroke reduction remains variable across studies [59,60].

When compared with the broader AngioVac experience, primarily used for right-heart and venous indications, the success rate observed here is consistent with published series and registry data reporting high rates of substantial thrombus/mass reduction, although some studies have reported substantial resource utilization, including transfusion requirements, vasopressor support, and intensive care admission [61,62,63]. This comparison is useful because it suggests that the extracorporeal suction–filtration platform can be capable of achieving substantial mass reduction across different anatomic settings; however, left-sided anatomy introduces distinct hazards, including systemic embolization, valvular trauma, and hemodynamic deterioration, which may not be fully reflected in aggregate AngioVac datasets dominated by right-sided disease. The small but serious adverse events summarized in this review (perforation, progressive MR/PVL, fatal deterioration) therefore remain highly consequential and argue for standardized definitions and outcome reporting.

Finally, the sizable proportion of non-infected masses, including left atrial appendage-related thrombus scenarios, suggests an expanding potential niche for aspiration thrombectomy when anticoagulation is ineffective or contraindicated and surgery is not an option. Recent reports specifically evaluating aspiration thrombectomy for persistent LAA thrombus and atrial thrombus extraction further support feasibility in carefully selected patients, often framed explicitly as a last-resort strategy when conventional options fail [10,64]. Based on the available literature, the most appropriate role for left-sided AngioVac debulking appears to be in carefully selected patients who are poor surgical candidates, require reduction of embolic burden, or need bridging to definitive surgical intervention. Going forward, the evidentiary priority is not additional isolated case reports but rather prospective multicentre registries (or pragmatic trials where feasible) with harmonized definitions (technical success, neurologic events, valve injury, infection control, need for subsequent surgery, and survival) and minimum follow-up windows. Until such data exist, left-sided AngioVac debulking should remain a highly selected, team-based intervention used with transparent acknowledgment of uncertainty, explicit documentation of why guideline-standard surgery is not pursued, and robust post-procedure surveillance.

### 4.2. Risk Factors for Procedure-Related Complications

Clinically significant complications occurred in 5/42 patients (11.9%), and their distribution across procedural and clinical characteristics was not uniform (Table 5). The strongest signal related to access route. All three mechanical complications, namely the paravalvular leak [28], the progressive mitral regurgitation preceding late death [40] and the cardiac perforation requiring emergency surgery [44], occurred in patients treated transapically, giving a complication rate of 3/8 (37.5%) for transapical access versus 0/29 (0%) for transseptal access (*p* = 0.007, Fisher’s exact test); the two remaining patients with complications had access route not reported [48,52]. A mechanistic explanation is plausible, in that transapical delivery provides a short, direct and relatively rigid trajectory to the mitral apparatus that transmits force to the subvalvular structures and the ventricular wall with limited ability to reorient the cannula, whereas the transseptal route approaches the valve along a more compliant curve.

The second signal related to the causative organism. Both patients with fungal endocarditis, one due to *Histoplasma capsulatum* [48] and one due to *Candida parapsilosis* [52], sustained a major adverse event, compared with 3/40 (7.5%) of all other patients (*p* = 0.012); one died of recurrent embolism and septic shock and the other sustained multiple cerebral infarctions and was left with residual mass. This is biologically coherent, since fungal vegetations are characteristically large, friable and prone to fragmentation, and fungal endocarditis carries a high embolic burden and high mortality even with combined medical and surgical therapy, while debulking neither eradicates the organism nor addresses perivalvular extension.

Prosthetic material showed a weaker, non-significant association, with complications in 3/10 patients (30.0%) with prosthetic valve involvement versus 2/32 (6.3%) without (*p* = 0.24). This is consistent with the mechanical difficulty of aspirating material adherent to a rigid sewing ring or occluder without disrupting the prosthesis or its seal, and it is notable that the single paravalvular leak arose in a patient with combined mitral and aortic prostheses [28]. By contrast, mass size showed no relationship with complications: among patients with a complication and a reported mass size the mean was 24.5 mm, close to the cohort mean of 23.3 mm, and complications occurred in 3/14 (21.4%) of masses ≥ 20 mm versus 1/9 (11.1%) of smaller masses (*p* = 1.00). Neither of the two largest masses, at 55 mm and 57 mm, was associated with an adverse event.

Finally, cerebral embolic protection showed a direction of effect consistent with benefit but did not reach significance: no new cerebrovascular event was reported among the 26 protected patients, versus 2/16 among the unprotected (*p* = 0.14). Both events occurred in unprotected patients, and filter-capture data from transcatheter aortic valve replacement consistently demonstrate debris liberation during left-heart instrumentation [59,60].

### 4.3. Relationship to Previously Published Syntheses

Three prior syntheses have addressed AngioVac use, and the present review differs from each in scope rather than merely in recency. The meta-analysis by Hameed et al. [19] pooled studies of AngioVac extraction of venous thromboses and endocardial vegetations and reported a pooled successful-removal rate of 74.5% (95% CI 48.2–90.2) among vegetation patients and 80.5% (95% CI 70.0–88.0) among those with right atrial or caval thrombi, alongside a pooled operative mortality of 14.6% and conversion to open surgery in 25.0% of vegetation patients; its cohort was nonetheless overwhelmingly venous and right-sided, and left-sided intracardiac targets were too few to be analysed separately. The systematic review by Enezate et al. [20] similarly spanned intravascular and intracardiac masses at all anatomical sites and reported high aggregate technical success, again without a left-sided subgroup analysis. The scoping review by Alshair et al. [22] was restricted by design to right-sided infective endocarditis. More recently, the multidisciplinary summit proceedings and technical review of percutaneous mechanical aspiration in infective endocarditis [65,66] have provided expert consensus, including explicit caution regarding left-sided application, but were narrative rather than systematic and did not assemble patient-level data.

The contribution of the present review is therefore not the extension of the search window alone. It is the assembly of a left-sided-only, patient-level dataset in which effectiveness is adjudicated against a single pre-specified threshold with the underlying evidence tier made explicit, in which infective and non-infective aetiologies are reported separately, in which outcomes are stratified by time and by attributability, and in which the risk of patient duplication is quantified rather than assumed away. That said, the technical success of 83.3% observed here is broadly comparable to the pooled estimates from predominantly right-sided series. It is at least as consistent with a shared reporting bias favouring successful cases across the whole AngioVac literature as it is with genuine equivalence of left- and right-sided performance. The distinguishing feature of the left-sided experience is not a lower success rate but a different complication profile, in which systemic rather than pulmonary embolization, valvular injury and cardiac perforation predominate.

A comparison with prospectively collected data reinforces this caution and, in our view, should govern how the headline figure of this review is read. In the multicentre RAPID registry, which prospectively enrolled 234 patients at 21 sites, removal of 70–100% of the target was achieved in only 58.5% of patients with right heart masses and 73.6% of those with caval thromboemboli [67], which is substantially below both the 83.3% technical success observed in the present left-sided cohort and the 74.5% pooled estimate from retrospective vegetation series [19]. It is implausible that left-sided debulking is genuinely more effective than right-sided debulking, given the more demanding access, the smaller working space and the greater consequence of embolization. The more parsimonious explanation is study design: prospective consecutive enrolment captures the failures that case reports do not. The same asymmetry applies to safety, since the pooled operative mortality of 14.6% and conversion rate of 25.0% reported for vegetation patients [19] exceed the 2.4% in-hospital mortality and 2.4% conversion observed here by a wide margin.

### 4.4. Study Limitations

The evidence base is dominated by case reports and very small series, making publication and selection bias highly likely, as successful or novel procedures are more likely to be reported than unsuccessful interventions or procedures complicated by adverse outcomes. Additional limitations include heterogeneous anatomic targets and techniques, inconsistent reporting of mass characteristics, embolic protection use, and follow-up, and variable definitions for outcomes and complications. Only around half of the studies reported mass size, and even fewer reported length of stay, limiting precision for key secondary endpoints. Because of non-uniform reporting and small sample sizes, meta-analysis was not appropriate, and the true rates of embolic events, valve injury, and longer-term recurrence or need for surgery remain uncertain. Furthermore, infected vegetations, thrombotic lesions, and other non-infectious masses represent biologically distinct entities with substantially different procedural goals, embolic risks, and expected outcomes. Pooling these lesions limits disease specific interpretation.

Several limitations specific to the conduct of this review should also be acknowledged. Screening, full-text eligibility assessment and adjudication of technical success were performed independently by two reviewers (F.B. and G.S.), with disagreements resolved by consensus; data extraction was performed by one reviewer and verified by the second, so a residual risk of extraction error cannot be fully excluded. The 70% threshold used to define technical success is pragmatic and has not been validated against clinical outcomes. Because 23 of 30 reports (76.7%) provided only qualitative imaging or narrative operator assessment (Section 3.5), the pooled success estimate is operator-reported rather than independently verified. Patient overlap between reports from the same institutions could not be definitively excluded, although the sensitivity analyses in Section 3.6 indicate that plausible duplication does not materially alter any principal estimate. One patient underwent extraction of an aortic arch mass that is not strictly intracardiac; this patient is retained in the primary analysis for transparency and excluded in sensitivity analysis. Non-English publications were excluded and no grey literature or trial registry search was undertaken, both of which may compound publication bias. Finally, the risk-factor analyses presented in Section 4.2 are exploratory, unadjusted and based on very small event counts; they identify signals for prospective testing and do not establish causation. Most importantly, the comparison with prospective registry data presented in Section 4.3 indicates that the effectiveness estimate reported here is best regarded as an upper bound rather than a central estimate.

These limitations converge on a single recommendation, namely that future reports adopt a minimum reporting dataset. On the basis of the gaps identified here, that dataset should include a calculated operative risk score together with the explicit reason surgery was declined; the presence and type of intracardiac prosthetic material and of any cardiac implantable electronic device; pre- and post-procedural mass dimensions measured by a stated modality; the use and type of cerebral embolic protection; systematic post-procedural neuroimaging and neurological assessment irrespective of symptoms; a time-stratified complication account distinguishing intraprocedural, in-hospital or 30-day, and late events; independent adjudication of whether each event was procedure-related; the occurrence and timing of any subsequent surgical or transcatheter intervention; and all-cause mortality at fixed intervals, with an explicit account of patients lost to follow-up. Prospective multicentre registry data collected to this standard, rather than further isolated case reports, will determine whether left-sided debulking has a durable clinical role.

## 5. Conclusions

This review provides the first left-sided-only, patient-level synthesis of percutaneous vacuum-assisted debulking, in which effectiveness was adjudicated against a single pre-specified threshold, infective and non-infective lesions were analysed separately, and the risk of duplicate publication was quantified rather than assumed absent. Three findings emerge that were not visible in earlier, anatomically mixed syntheses: every mechanical complication followed transapical access and none followed a transseptal approach; both patients with fungal endocarditis sustained a major adverse event, as did a higher proportion of those with prosthetic valve material; and cerebral embolic protection was deployed according to institutional practice rather than mass size, with each reported post-procedural embolic event occurring in an unprotected patient. Reported technical success nevertheless remains operator-adjudicated, exceeds that of the only prospective registry of this device, and did not preclude death. Left-sided debulking is therefore best positioned as a bridge or palliative strategy rather than a substitute for guideline-indicated surgery, with transseptal access and embolic protection preferred and particular caution where the aetiology is fungal or prosthetic material is involved; defining its role will require prospective multicentre registries rather than further isolated case reports.

## Figures and Tables

**Table 1:** Characteristics of the studies included in the review.

Study Number	Authors	Year	Country	Study Design
1	Bansal et al. [26]	2024	USA	Case Report
2	Frisoli et al. [27]	2022	USA	Case Report
3	Fiocco et al. [28]	2023	Italy	Case Series
4	Ramanan TT [29]	2020	USA	Abstract
5	Gerosa et al. [30]	2020	Italy	Case Report
6	Memon et al. [31]	2022	USA	Case Report
7	Qintar et al. [32]	2022	USA	Case Series
8	Gerosa et al. [33]	2023	Italy	Case Report
9	Xu et al. [34]	2022	USA	Case Report
10	Brown et al. [35]	2023	NR	Case Report
11	Antoun et al. [36]	2022	NR	Case Report
12	Eilers et al. [37]	2022	USA	Case Report
13	So et al. [38]	NR	USA	Case Report
14	Knipe et al. [39]	2022	NR	Case Report
15	Rabenstein et al. [40]	2022	USA	Case Report
16	Lane et al. [41]	2023	USA	Case Report
17	Gunga et al. [42]	2024	Switzerland	Case Report
18	Ahmed et al. [43]	2022	USA	Case Report
19	Tarzia et al. [44]	2023	Italy	Case Series
20	Kucuk et al. [45]	2023	USA	Case Report
21	Ashukem et al. [46]	2019	USA	Case Report
22	Cioci et al. [47]	2024	USA	Case Report
23	Qafisheh et al. [48]	2025	USA	Case Report
24	Al Asmari et al. [49]	2025	Canada	Case Report
25	Amar et al. [50]	2025	USA	Case Report
26	Verghese et al. [51]	2025	USA	Case Report
27	Affas et al. [52]	2025	Qatar	Case Report
28	Massa et al. [53]	2024	USA	Abstract
29	Cao et al. [54]	2024	USA	Abstract
30	Abdalla et al. [55]	2025	USA	Abstract

NR—Not Reported.

**Table 2:** Patient characteristics.

Study	Number of Patients	Age (Years)	Sex	IE	Pathogen
Bansal et al. [26]	1	66	Female	Yes	*Staphylococcus* *lugdunensis*
Frisoli et al. [27]	1	77	Male	No	N/A
Fiocco et al. [28]	2	57, 54	Female, Male	Yes (1/2)	*Enterococcus faecalis*
Ramanan TT [29]	1	86	Female	Yes	*Streptococcus gordonii*
Gerosa et al. [30]	1	70	Female	Yes	*Staphylococcus* *epidermidis*
Memon et al. [31]	1	73	Female	Yes	MSSA
Qintar et al. [32]	10	Mean 58.3 (±17.3)	5 females, 5 males	No	N/A
Gerosa et al. [33]	1	68	Female	No	N/A
Xu et al. [34]	1	49	Female	Yes	Culture-negative
Brown et al. [35]	1	76	Male	Yes	MRSA
Antoun et al. [36]	1	31	Male	Yes	MSSA
Eilers et al. [37]	1	41	Female	Yes	*Staphylococcus* *lugdunensis*
So et al. [38]	1	63	Female	No	N/A
Knipe et al. [39]	1	82	Female	Yes	*Staphylococcus epidermidis*
Rabenstein et al. [40]	1	79	Male	Yes	NR
Lane et al. [41]	1	57	Male	Yes	NR
Gunga et al. [42]	1	54	Male	No	N/A
Ahmed et al. [43]	1	76	Male	Yes	MRSA
Tarzia et al. [44]	3	Mean 58.5 (±16.5)	NR	Yes (3/3)	NR
Kucuk et al. [45]	1	68	Female	No	N/A
Ashukem et al. [46]	1	65	Male	No	N/A
Cioci et al. [47]	1	75	Male	Yes	*Cardiobacterium hominis*
Qafisheh et al. [48]	1	76	Female	Yes	*Histoplasma capsulatum*
Al Asmari et al. [49]	1	81	Male	Yes	*Streptococcus mutans*
Amar et al. [50]	1	84	Female	No	N/A
Verghese et al. [51]	1	63	Female	Yes	*Enterococcus faecalis*
Affas et al. [52]	1	50	Male	Yes	*Candida parapsilosis*
Massa et al. [53]	1	30	Female	Yes	*Staphylococcus aureus*
Cao et al. [54]	1	68	Male	Yes	MSSA
Abdalla et al. [55]	1	NR	NR	No	N/A

IE, infective endocarditis; MSSA, methicillin-susceptible *Staphylococcus aureus*; MRSA, methicillin-resistant *Staphylococcus aureus*; N/A, not applicable; and NR, not reported. “Yes (1/2)” indicates that infective endocarditis was present in one of the two patients reported by Fiocco et al. [28]; the other patient underwent debulking of a non-infected mass.

**Table 3:** Procedural details.

Study	Number of Patients	Access Route	Mass Location	Mass Size (mm)	Cerebral Protection Device
Bansal et al. [26]	1	Transseptal via femoral veins	MV posterior leaflet	33 mm	SENTINEL™
Frisoli et al. [27]	1	Transseptal via femoral veins	LAA closure device	22 mm	SENTINEL™
Fiocco et al. [28]	2	Transapical via mini-thoracotomy	MV leaflet; MV and AV prostheses	20 mm	TriGuard
Ramanan TT [29]	1	Transseptal (modified approach)	MV posterior leaflet	11 mm	SENTINEL™
Gerosa et al. [30]	1	Transapical via mini-thoracotomy	MV bioprosthesis	20 mm	NR
Memon et al. [31]	1	Transseptal via femoral veins	MV posterior-medial aspect	NR	SENTINEL™
Qintar et al. [32]	10	Transseptal, and modified approach	LA, LAA, MV, aortic arch	NR	SENTINEL™
Gerosa et al. [33]	1	Transseptal (modified approach)	MV leaflet	20 mm	NR
Xu et al. [34]	1	Transseptal via femoral veins	MV prosthesis	NR	SENTINEL™
Brown et al. [35]	1	Transseptal (modified approach)	MV leaflet	12 mm	NR
Antoun et al. [36]	1	Transseptal via femoral veins	MV leaflet	21 mm	SENTINEL™
Eilers et al. [37]	1	Transseptal via femoral veins	MV, LA, SVC baffle	NR	NR
So et al. [38]	1	Transseptal via femoral veins	LAA	NR	Watchman device
Knipe et al. [39]	1	Transseptal (modified approach)	MV leaflet	NR	NR
Rabenstein et al. [40]	1	Transapical via mini-thoracotomy	Anterior MV leaflet	NR	SENTINEL™
Lane et al. [41]	1	Transseptal via femoral veins	Posterior MV leaflet	NR	SpiderFX
Gunga et al. [42]	1	Transapical via mini-thoracotomy	Mitral bioprosthesis	18 mm	SENTINEL™
Ahmed et al. [43]	1	Transseptal via femoral veins	Anterior MV leaflet	NR	NR
Tarzia et al. [44]	3	Transapical via mini-thoracotomy	Mitral valve	23 mm	NR
Kucuk et al. [45]	1	Transseptal via femoral veins	Posterolateral MV annulus	26 mm	SENTINEL™
Ashukem et al. [46]	1	Transseptal via femoral veins	LA and prosthetic MV	57 mm	NR
Cioci et al. [47]	1	Retrograde arterial access via femoral artery	Aortic valve	18 mm	NR
Qafisheh et al. [48]	1	NR	Aortic valve	40 mm	NR
Al Asmari et al. [49]	1	Transseptal via femoral veins	Mitral bioprosthesis	15 mm	SENTINEL™
Amar et al. [50]	1	Transseptal via femoral veins	Mitral bioprosthesis	55 mm	TriGuard
Verghese et al. [51]	1	Transseptal via femoral veins	Mitral bioprosthesis	12 mm	NR
Affas et al. [52]	1	NR	Mitral bioprosthesis	15 mm	NR
Massa et al. [53]	1	NR	MV, spanning the annulus	15 mm	NR
Cao et al. [54]	1	Transseptal (percutaneous)	Posterior MV leaflet	18 mm	Device used, type NR
Abdalla et al. [55]	1	NR	NR	NR	NR

AV, aortic valve; LA, left atrium; LAA, left atrial appendage; MV, mitral valve; NR, not reported; and SVC, superior vena cava.

**Table 4:** Patient outcomes.

Study	Number of Patients	Debulking Outcome	Post-Procedure Complications	Length of Hospital Stay (Days)	Follow-Up Duration
Bansal et al. [26]	1	Successful debulking	None	NR	NR
Frisoli et al. [27]	1	Residual mass	None	2 days	1 month
Fiocco et al. [28]	2	Successful debulking	1 patient had PVL at 1 month	NR	1 week to 6 months
Ramanan TT [29]	1	Successful debulking	None	4 days	NR
Gerosa et al. [30]	1	Successful debulking	None	NR	NR
Memon et al. [31]	1	Successful debulking	None	3 days	NR
Qintar et al. [32]	10	Successful debulking in 80% of cases	None	NR	1–16 months
Gerosa et al. [33]	1	Successful debulking	None	NR	NR
Xu et al. [34]	1	Successful debulking	None	NR	6 weeks
Brown et al. [35]	1	Residual mass	None	NR	NR
Antoun et al. [36]	1	Successful debulking	None	NR	NR
Eilers et al. [37]	1	Successful debulking	None	NR	NR
So et al. [38]	1	Successful debulking	None	NR	NR
Knipe et al. [39]	1	Successful debulking	None	NR	6 weeks
Rabenstein et al. [40]	1	Successful debulking	Progressive MR over months; patient died at 4 months	30 days	4 months
Lane et al. [41]	1	Successful debulking	None	30 days	6 weeks
Gunga et al. [42]	1	Successful debulking	None	NR	1 year
Ahmed et al. [43]	1	Successful debulking	None	NR	NR
Tarzia et al. [44]	3	Successful debulking	Cardiac perforation requiring emergent open heart surgery	NR	Median 2.5 years
Kucuk et al. [45]	1	Successful debulking	None	1 day	NR
Ashukem et al. [46]	1	Residual mass	None	NR	NR
Cioci et al. [47]	1	Successful debulking	None	1 day	5 months
Qafisheh et al. [48]	1	Successful debulking	Recurrent embolic events, septic shock, death	NR	NR
Al Asmari et al. [49]	1	Successful debulking	None	NR	30 months
Amar et al. [50]	1	Successful debulking	None	NR	NR
Verghese et al. [51]	1	Successful debulking	None	12 days	6 weeks
Affas et al. [52]	1	Residual mass	Multiple cerebral infarctions	NR	6 weeks
Massa et al. [53]	1	Successful debulking	None	NR	NR
Cao et al. [54]	1	Successful debulking	None	NR	NR
Abdalla et al. [55]	1	Successful debulking	None	NR	NR

MR, mitral regurgitation; NR, not reported; and PVL, paravalvular leak. Successful debulking was defined as removal of at least 70% of the mass size.

**Table 5:** Exploratory analysis of candidate risk factors for clinically significant procedure-related complications.

Candidate Risk Factor	Complication Rate	Comparator	*p* Value
Transapical access	3/8 (37.5%)	Transseptal 0/29 (0%)	0.007
Fungal aetiology	2/2 (100%)	Non-fungal 3/40 (7.5%)	0.012
Prosthetic valve involvement	3/10 (30.0%)	No prosthesis 2/32 (6.3%)	0.24
No cerebral embolic protection (new cerebrovascular event)	2/16 (12.5%)	Protection documented 0/26 (0%)	0.14
Mass ≥ 20 mm	3/14 (21.4%)	Mass < 20 mm 1/9 (11.1%)	1.00

*p* values are two-sided and derived from Fisher’s exact test. Mass-size comparisons are restricted to the 23 patients with a reported mass size. All analyses are exploratory, unadjusted for multiple comparisons or confounding by indication, and hypothesis-generating only.

## Data Availability

No new data were created or analyzed in this study. Data sharing is not applicable to this article.

## Supplementary Materials

The following supporting information can be downloaded at: https://www.mdpi.com/article/10.3390/jcm15176669/s1, Table S1: PRISMA 2020 Checklist.

## References

[1] Bussani R., Castrichini M., Restivo L., Fabris E., Porcari A., Ferro F., Pivetta A., Korcova R., Cappelletto C., Manca P. (2020). Cardiac Tumors: Diagnosis, Prognosis, and Treatment. Curr. Cardiol. Rep..

[2] Desch S., Freund A., de Waha S., Eitel I., Lurz P., Stiermaier T., Fuernau G., Schuler G., Thiele H. (2014). Outcome in patients with left-sided native-valve infective endocarditis and isolated large vegetations. Clin. Cardiol..

[3] Hoffmeier A., Sindermann J.R., Scheld H.H., Martens S. (2014). Cardiac tumors--diagnosis and surgical treatment. Dtsch. Ärztebl. Int..

[4] Scheggi V., Bohbot Y., Tribouilloy C., Trojette F., Di Lena C., Philip M., Hubert S., Menale S., Zoppetti N., Del Pace S. (2023). Impact of cardiac surgery on left-sided infective endocarditis with intermediate-length vegetations. Heart.

[5] Bohbot Y., Habib G., Laroche C., Stöhr E., Chirouze C., Hernandez-Meneses M., Melissopoulou M., Mutlu B., Scheggi V., Branco L. (2022). Characteristics, management, and outcomes of patients with left-sided infective endocarditis complicated by heart failure: A substudy of the ESC-EORP EURO-ENDO (European infective endocarditis) registry. Eur. J. Heart Fail..

[6] Habib G., Erba P.A., Iung B., Donal E., Cosyns B., Laroche C., Popescu B.A., Prendergast B., Tornos P., Sadeghpour A. (2019). Clinical presentation, aetiology and outcome of infective endocarditis. Results of the ESC-EORP EURO-ENDO (European infective endocarditis) registry: A prospective cohort study. Eur. Heart J..

[7] Mohananey D., Mohadjer A., Pettersson G., Navia J., Gordon S., Shrestha N., Grimm R.A., Rodriguez L.L., Griffin B.P., Desai M.Y. (2018). Association of Vegetation Size With Embolic Risk in Patients With Infective Endocarditis: A Systematic Review and Meta-analysis. JAMA Intern. Med..

[8] Papadimitriou-Olivgeris M., Guery B., Ianculescu N., Auberson D., Tozzi P., Kirsch M., Monney P. (2024). Risk of embolic events before and after antibiotic treatment initiation among patients with left-side infective endocarditis. Infection.

[9] de Miguel M., López J., Vilacosta I., Olmos C., Sáez C., Cabezón G., Zulet P., Jerónimo A., Gómez D., Pulido P. (2024). Clinical Profile and Prognosis of Patients with Left-Sided Infective Endocarditis with Surgical Indication Who Are Not Operated. Microorganisms.

[10] Azpiroz-Franch M.J., Rello-Sabaté P., Oristrell-Santamaría G., González-Alujas T., Martí-Aguasca G., Soriano-Colomé T. (2021). Atrial thrombus aspiration through the AngioVac system: An alternative when surgery and anticoagulation are not an option. Rev. Esp. Cardiol. Engl. Ed..

[11] Almanfi A., Nabous I. (2022). Percutaneous Aspiration of Vegetation in Tricuspid Valve Infective Endocarditis: A Possible Novel Treatment Option. JACC Case Rep..

[12] Keeton J., Gonzalez P.E., Cox J., Morlend R.M., Amin A.A., Mammen P.P.A., Huffman L.C., Araj F.G. (2020). Saved by the VAC: Minimally Invasive Removal of a Surely Fatal Right Heart Thrombus in a Patient with Advanced Heart Failure. Case Rep. Cardiol..

[13] Lin C.Y., Singhal A.K., Cavanaugh N.B., Subramani S., Bang J.K., Hanada S. (2024). AngioVac Procedures: Integration of cardiac surgeon and anesthesiologist-led transesophageal echocardiography: A preliminary report. Heliyon.

[14] Thiagaraj A.K., Malviya M., Htun W.W., Telila T., Lerner S.A., Elder M.D., Schreiber T.L. (2017). A Novel Approach in the Management of right-sided Endocarditis: Percutaneous Vegectomy Using the AngioVac Cannula. Future Cardiol..

[15] Mittal N., Mittal R., Ramon M.C., Sly Z., Ansari M.M., Ramon M. (2022). A Novel Technique Debulking Vegetations in Tricuspid Endocarditis and Venacava Utilizing AngioVac Aspiration System. Cureus.

[16] Haupt B., Merkle F., Dreizler T., Falk V., Starck C. (2021). Technical implementation of percutaneous thrombus aspiration using the AngioVac system. Perfusion.

[17] Nickell A., Sergev O., Alberto N., Bande D., Guerrero D.M. (2023). Effectiveness of the vacuum assisted aspiration AngioVac system in the removal of intravascular masses. Catheter. Cardiovasc. Interv..

[18] Dandu C., Alamzaib S.M., Patel D., Naughton R., Polina A., Najam M., Alhusain R., Patel N., Sattar Y., Alraies M.C. (2023). Investigating the Complications and Causes of Failure of the AngioVac System: A Post-Marketing Surveillance From the MAUDE Database. Cureus.

[19] Hameed I., Lau C., Khan F.M., Wingo M., Rahouma M., Leonard J.R., Di Franco A., Worku B.M., Salemi A., Girardi L.N. (2019). AngioVac for extraction of venous thromboses and endocardial vegetations: A meta-analysis. J. Card. Surg..

[20] Enezate T., Alkhatib D., Raja J., Chinta V., Patel M., Omran J. (2022). AngioVac for Minimally Invasive Removal of Intravascular and Intracardiac Masses: A Systematic Review. Curr. Cardiol. Rep..

[21] Pawlichuk D., Pippin M. (2024). A Case of Right-Sided Infective Endocarditis Requiring AngioVac Debulking. Cureus.

[22] Alshair F.M., Alsulami A.S., Baghaffar A.H., Fatani M.A. (2024). A novel approach, AngioVac use in right-sided infective endocarditis: A scoping review. Cardiothorac. Surg..

[23] Page M.J., McKenzie J.E., Bossuyt P.M., Boutron I., Hoffmann T.C., Mulrow C.D., Shamseer L., Tetzlaff J.M., Akl E.A., Brennan S.E. (2021). The PRISMA 2020 statement: An updated guideline for reporting systematic reviews. BMJ.

[24] Stang A. (2010). Critical evaluation of the Newcastle-Ottawa scale for the assessment of the quality of nonrandomized studies in meta-analyses. Eur. J. Epidemiol..

[25] Munn Z., Barker T.H., Moola S., Tufanaru C., Stern C., McArthur A., Stephenson M., Aromataris E. (2020). Methodological quality of case series studies: An introduction to the JBI critical appraisal tool. JBI Evid. Synth..

[26] Bansal N., Younes S., Maaieh M. (2024). Percutaneous Debulking of a Large Mobile Mitral Valve Vegetation Using the Angiovac. Cureus.

[27] Frisoli T.M., Chiang M., Eng M.H., Gonzalez P.E., Szymanski T., Villablanca P.A., O’Neill B., Lee J.C., Wang D.D., O’Neill W.W. (2022). Percutaneous Aspiration Thrombectomy of Thrombus Attached to Left Atrial Surface of a Watchman FLX Device. JACC Clin. Electrophysiol..

[28] Fiocco A., Colli A., Besola L. (2023). Case report: Treatment of left-sided valve endocarditis using the Transapical AngioVac System and cerebral embolism protection device: A case series. Front. Cardiovasc. Med..

[29] Ramanan T.T. Trans-Septal AngioVac Debulking of Mitral Valve Endocarditis [Abstract]. Proceedings of the Scientific Sessions of the Society for Cardiovascular Angiography and Interventions (SCAI).

[30] Gerosa G., Longinotti L., Bagozzi L., D’Onofrio A., Zanella F., Pittarello D., Tarzia V. (2020). Transapical Aspiration of a Mitral Mass With the AngioVac System on a Beating Heart. Ann. Thorac. Surg..

[31] Memon S., Goldman S., Hawthorne K.M., Gnall E.M. (2022). Percutaneous transeptal mitral valve endocarditis debulking with AngioVac aspiration system. Catheter. Cardiovasc. Interv..

[32] Qintar M., Wang D.D., Lee J., Villablanca P., Eng M.H., Frisoli T., O’Neill B.P., O’Neill W.W. (2022). Transcatheter vacuum-assisted left-sided mass extraction with the AngioVac system. Catheter. Cardiovasc. Interv..

[33] Gerosa G., Ponzoni M., Evangelista G., Tessari C., Tiberio I., Molè A., Zanella F., Pittarello D., Tarzia V. (2023). Proof of Concept: Trans-atrial AngioVac Aspiration of Mitral Valve Thrombosis in a COVID-19 Patient. ASAIO J..

[34] Xu J., Turaga S., Bhama J., Vallurupalli S., Dhar G. (2022). Percutaneous Aspiration (AngioVac) of a Mitral Valve Vegetation followed by a Transcatheter Mitral Valve-in-Valve Intervention. Methodist DeBakey Cardiovasc. J..

[35] Brown T., Stoner B., Kundu A. (2023). Successful Use of AngioVac for Debulking of Mitral Valve Endocarditis. J. Am. Coll. Cardiol..

[36] Antoun M., Jafar N.L., Qintar M., Edward S.O., Moore S.R., Qandeel H.G., Mallory M.A., Jones T.N., Wildern W. (2022). Vacuum to the Rescue: Trans-Septal Angiovac for Mitral Valve Endocarditis. Chest.

[37] Eilers L.F., Gowda S.T., Lam W.W., Parekh D.R. (2022). Use of AngioVac Vacuum Aspiration for Refractory Endocarditis in a Postoperative Mustard Patient. Tex. Heart Inst. J..

[38] So C.Y., Wang D.D., Kang G., Villablanca P.A., Frisoli T., O’Neill W.W. (2020). Vacuuming the LAA: Left Atrial Appendage Thrombectomy Using AngioVac to Facilitate Percutaneous Mitral Balloon Valvuloplasty. Struct. Heart.

[39] Knipe J., Mohabir S.N., Girard M.J., Patton M. (2022). A case of trans-septal percutaneous aspiration via AngioVac in a patient with a large mitral valve septic vegetation presenting with embolic ischemic stroke. Chest.

[40] Rabenstein A.P., Khalif A., Lasorda D., Popeck M., Chakravarthy M., McGregor W. (2022). Novel use of a transapical endovascular suction device for mitral valve endocarditis in a high-risk surgical patient with successful cerebral protection. JTCVS Tech.

[41] Lane C.M., El Sabbagh A., Alkhouli M.A. (2023). Transseptal Debulking of Mitral Valve Endocarditis Using On-Circuit Aspiration Thrombectomy System and Multimodal Embolic Protection. JACC Cardiovasc. Interv..

[42] Gunga Z., Rubimbura V., Oberson D., Monney P., Bechtold X., Ltaief Z., Rancati V., Eeckhout E., Kirsch M. (2024). Thromboaspiration of a left-sided bioprosthetic valve thrombosis by a mini-access: The Lausanne novel procedure. Front. Cardiovasc. Med..

[43] Ahmed T., Hawamdeh H.F., Al-Abdouh A., Kotter J.R., Messerli A.W., Gurley J.C. (2022). A novel approach for debulking mitral valve infective endocarditis; why swim against the current?. Curr. Probl. Cardiol..

[44] Tarzia V., Ponzoni M., Tessari C., Evangelista G., Zanella F., Pittarello D., Gerosa G. (2023). Navigating the Heart. The Evolution of the AngioVac System in a Single-center Experience. Curr. Probl. Cardiol..

[45] Kucuk H.O., Nan J.Z., Larson K.F., Sinak L.J., Eleid M.F. (2023). Percutaneous Vacuum-Assisted Aspiration of Mobile Caseous Mitral Annulus Calcification. JACC Case Rep..

[46] Ashukem M., Seibolt L., Verma D.R., Loli A., Byrne T. (2019). A case of trans-septal left atrial thrombectomy utilizing AngioVac extracorporeal venous-venous cardiopulmonary bypass filter circuit in a patient with obstructive shock from large LA and prosthetic valve thrombosis post TMVR. J. Am. Coll. Cardiol..

[47] Cioci A.L., Hutton A., Yi S., Khoriaty G., Gottlieb G., Cartledge R., Rubenstein M. (2024). Percutaneous aspiration of aortic valve vegetation in a patient with aortic valve endocarditis. Clin. Case Rep..

[48] Qafisheh Q., Shubietah A., Aljunaidi R., Sajdeya O., Baniowda M.A., Giesige C., El-Hajj S.C., Grande R. (2025). AngioVac-assisted management of histoplasma capsulatum endocarditis in a bioprosthetic aortic valve: Challenges and outcomes. J. Cardiothorac. Surg..

[49] Al Asmari A., Chan K.L. (2025). Percutaneous Debulking of Bioprosthetic Mitral Valve Vegetations: Preserving Valve Function in Recurrent Infective Endocarditis. JACC Case Rep..

[50] Amar G., Patail H., Spevack D., Haidry S., Agarwal A., Lansman S., Ohira S., Spielvogel D., Ahmad H., Shimamura J. (2025). Transcatheter Vacuum-Assisted Mass Extraction on a Mitral Bioprosthesis. JACC Case Rep..

[51] Verghese D., Navas V., Wang D.D., Howard T., Paz L.H., Solomon B., Axline D., Cudemus G., Albaghdadi M., Cubeddu R.J. (2025). Heart and Brain Team Salvaging the Mechanical Mitral Valve: Step-by-Step Percutaneous Aspiration of Mechanical Mitral Valve Endocarditis. JACC Case Rep..

[52] Affas M.N., Chawa Y., Khalil M.S., Alkodmani S. (2024). Cardioembolic Stroke Due to Prosthetic Valve Endocarditis Caused by Candida parapsilosis: A Case Report. Case Rep. Infect. Dis..

[53] Massa B., Girard M.J., Patton M., Perez V., James C. (2024). The use of the AngioVac system for the treatment of left-sided endocarditis. Chest.

[54] Cao J.J.L., Rihal C., Motiei A., Kietselaer B. (2024). Percutaneous aspiration of mitral valve endocarditis using the AngioVac device. Circulation.

[55] Abdalla M., Bhamber T., Anjum M., Reddy V., Telleria O., Min K. (2025). Left-sided AngioVac for the treatment of marantic endocarditis: A novel approach for a rare disease entity. Chest.

[56] Delgado V., Ajmone Marsan N., de Waha S., Bonaros N., Brida M., Burri H., Caselli S., Doenst T., Ederhy S., Erba P.A. (2023). 2023 ESC Guidelines for the management of endocarditis. Eur. Heart J..

[57] Kang D.H., Kim Y.J., Kim S.H., Sun B.J., Kim D.H., Yun S.C., Song J.M., Choo S.J., Chung C.H., Song J.K. (2012). Early surgery versus conventional treatment for infective endocarditis. N. Engl. J. Med..

[58] McDonald E.G., Aggrey G., Aslan A.T., Casias M., Cortes-Penfield N., Dong M.Q.D., Egbert S., Footer B., Isler B., King M. (2023). Guidelines for Diagnosis and Management of Infective Endocarditis in Adults: A WikiGuidelines Group Consensus Statement. JAMA Netw. Open.

[59] Kapadia S.R., Kodali S., Makkar R., Mehran R., Lazar R.M., Zivadinov R., Dwyer M.G., Jilaihawi H., Virmani R., Anwaruddin S. (2017). Protection Against Cerebral Embolism During Transcatheter Aortic Valve Replacement. J. Am. Coll. Cardiol..

[60] Kapadia S.R., Makkar R., Leon M., Abdel-Wahab M., Waggoner T., Massberg S., Rottbauer W., Horr S., Sondergaard L., Karha J. (2022). Cerebral Embolic Protection during Transcatheter Aortic-Valve Replacement. N. Engl. J. Med..

[61] Mullins J.B., Warner A., Patel V.S., Arora V. (2024). A Retrospective Analysis of AngioVac Outcomes at a Tertiary Care Center. J. Soc. Cardiovasc. Angiogr. Interv..

[62] Moriarty J.M., Liao M., Kim G.H.J., Yang E., Desai K., Ranade M., Plotnik A.N. (2022). Procedural outcomes associated with use of the AngioVac System for right heart thrombi: A safety report from RAPID registry data. Vasc. Med..

[63] Salsamendi J., Doshi M., Bhatia S., Bordegaray M., Arya R., Morton C., Narayanan G. (2015). Single Center Experience with the AngioVac Aspiration System. Cardiovasc. Interv. Radiol..

[64] Gianni C., Horton R.P., Nair G., La Fazia V.M., Mohanty S., Al-Ahmad A., Allison J.D., Bassiouny M.A., Bode W.D., Burkhardt J.D. (2025). Percutaneous Aspiration Thrombectomy in Patients With Persistent Left Atrial Appendage Thrombus: A Case Series. JACC Clin. Electrophysiol..

[65] Marinacci L.X., Sethi S.S., Paras M.L., El Sabbagh A., Secemsky E.A., Sohail M.R., Starck C., Bearnot B., Yucel E., Schaerf R.H.M. (2024). Percutaneous Mechanical Aspiration for Infective Endocarditis: Proceedings From an Inaugural Multidisciplinary Summit and Comprehensive Review. J. Soc. Cardiovasc. Angiogr. Interv..

[66] El Sabbagh A., Yucel E., Zlotnick D., Moriarty J.M., Younes S., Hamid N., Akhtar Y., Baddour L.M., O’Gara P., Starck C. (2024). Percutaneous Mechanical Aspiration in Infective Endocarditis: Applications, Technical Considerations, and Future Directions. J. Soc. Cardiovasc. Angiogr. Interv..

[67] Moriarty J.M., Rueda V., Liao M., Kim G.H.J., Rochon P.J., Zayed M.A., Lasorda D., Golowa Y.S., Shavelle D.M., Dexter D.J. (2021). Endovascular Removal of Thrombus and Right Heart Masses Using the AngioVac System: Results of 234 Patients from the Prospective, Multicenter Registry of AngioVac Procedures in Detail (RAPID). J. Vasc. Interv. Radiol..

