# Supplementary material for: Percutaneous Vacuum-Assisted Debulking of Infected and Non-Infected Left-Sided Cardiac Masses Using the AngioVac System: A Systematic Review of Published Case Reports and Case Series
Source: J Clin Med. 2026 Aug 28;15(17):6669. doi: 10.3390/jcm15176669 (PMC13566122; doi:10.3390/jcm15176669)
Supplement: Supplementary file 1 [file jcm-15-06669-s001.zip › jcm-4451946-supplementary.pdf]

PRISMA 2020 Checklist

Percutaneous Vacuum-Assisted Debulking of Infected and Non-Infected Left-Sided Cardiac Masses Using the AngioVac System: A Systematic Review

Bratosin F, DeSanctis J, Simeunovic G. • Submitted to Journal of Clinical Medicine (Manuscript ID: jcm-4451946). • Protocol registered on OSF (10.17605/OSF.IO/N384U). • PRISMA flow diagram: Figure 1.

Section and Topic	Item #	Checklist item	Location where item is reported
**TITLE**			
Title	1	Identify the report as a systematic review.	Title (p. 1): article type label “Systematic Review” and title “… Using the AngioVac System: A Systematic Review.”
**ABSTRACT**			
Abstract	2	See the PRISMA 2020 for Abstracts checklist.	Abstract (p. 1): structured abstract with Background and objectives, Methods, Results, and Conclusions.
**INTRODUCTION**			
Rationale	3	Describe the rationale for the review in the context of existing knowledge.	Section 1 (Introduction), paragraphs 1–4.
Objectives	4	Provide an explicit statement of the objective(s) or question(s) the review addresses.	Section 1 (Introduction), final paragraph (“This systematic review aims to …”); Abstract (Objectives).
**METHODS**			
Eligibility criteria	5	Specify the inclusion and exclusion criteria for the review and how studies were grouped for the syntheses.	Section 2.1 (Eligibility Criteria and Information Sources). Infected vs. non-infected masses grouped in Section 2.5 and Results.
Information sources	6	Specify all databases, registers, websites, organisations, reference lists and other sources searched or consulted to identify studies. Specify the date when each source was last searched or consulted.	Sections 2.1 and 2.2: PubMed, Scopus, and Web of Science; searched for reports published through November 2025.
Search strategy	7	Present the full search strategies for all databases, registers and websites, including any filters and limits used.	Section 2.2 (Search Strategy): full Boolean strings provided for PubMed, Scopus, and Web of Science; keywords/MeSH terms listed.
Selection process	8	Specify the methods used to decide whether a study met the inclusion criteria of the review, including how many reviewers screened each record and each report retrieved, whether they worked independently, and if applicable, details of automation tools used in the process.	Section 2.3 (Study Selection and Data Extraction): one reviewer (F.B.) screened titles/abstracts and assessed full texts (single reviewer; no automation tools used).
Data collection process	9	Specify the methods used to collect data from reports, including how many reviewers collected data from each report, whether they worked independently, any processes for obtaining or confirming data from study investigators, and if applicable, details of automation tools used in the process.	Section 2.3 (Study Selection and Data Extraction): data extracted by one reviewer (F.B.); no automation tools used.
Data items	10a	List and define all outcomes for which data were sought. Specify whether all results that were compatible with each outcome domain in each study were	Section 2.5 (Outcome Measures and Data Synthesis): primary effectiveness outcome (technical success, defined as ≥70% debulking without conversion) and

Section and Topic	Item #	Checklist item	Location where item is reported
		sought (e.g. for all measures, time points, analyses), and if not, the methods used to decide which results to collect.	primary safety outcome (procedure-related complications) defined.
Data items	10b	List and define all other variables for which data were sought (e.g. participant and intervention characteristics, funding sources). Describe any assumptions made about any missing or unclear information.	Section 2.5 (secondary outcomes: demographics, indication [IE vs. non-infected], microbiology, access route, target anatomy, embolic protection, mass size, length of stay, follow-up); Section 2.1 (population). No imputation was performed (Section 2.5).
Study risk of bias assessment	11	Specify the methods used to assess risk of bias in the included studies, including details of the tool(s) used, how many reviewers assessed each study and whether they worked independently, and if applicable, details of automation tools used in the process.	Section 2.4 (Quality Assessment and Risk of Bias): formal quality/risk-of-bias assessment was limited by the predominance of single-patient case reports; JBI critical-appraisal and Newcastle–Ottawa tools referenced [19,20]. A per-study risk-of-bias assessment was not formally applied.
Effect measures	12	Specify for each outcome the effect measure(s) (e.g. risk ratio, mean difference) used in the synthesis or presentation of results.	Section 2.5: results presented descriptively — categorical variables as counts and proportions; continuous variables as means and ranges. No pooled effect measures were computed (meta-analysis not conducted).
Synthesis methods	13a	Describe the processes used to decide which studies were eligible for each synthesis (e.g. tabulating the study intervention characteristics and comparing against the planned groups for each synthesis (item #5)).	Section 2.5 and Section 3.1 (Study selection): eligible studies tabulated and grouped (IE vs. non-infected masses) for descriptive synthesis.
	13b	Describe any methods required to prepare the data for presentation or synthesis, such as handling of missing summary statistics, or data conversions.	Section 2.5: individual patient values used when provided; study-reported aggregate means used otherwise; no imputation was performed for missing data.
	13c	Describe any methods used to tabulate or visually display results of individual studies and syntheses.	Section 2.5; results tabulated in Tables 1–4 and displayed graphically in Figures 2–3.
	13d	Describe any methods used to synthesize results and provide a rationale for the choice(s). If meta-analysis was performed, describe the model(s), method(s) to identify the presence and extent of statistical heterogeneity, and software package(s) used.	Section 2.5: qualitative (narrative) synthesis supported by descriptive statistics. Formal meta-analysis was not conducted, with rationale (predominance of case reports/small series, small samples, inconsistent variance and follow-up reporting).
	13e	Describe any methods used to explore possible causes of heterogeneity among study results (e.g. subgroup analysis, meta-regression).	Not applicable — no meta-analysis performed (Section 2.5). Infected vs. non-infected masses were described separately in a narrative subgroup manner.
	13f	Describe any sensitivity analyses conducted to assess robustness of the synthesized results.	Not applicable — no quantitative synthesis / sensitivity analyses performed (Section 2.5).
Reporting bias assessment	14	Describe any methods used to assess risk of bias due to missing results in a synthesis (arising from reporting biases).	No formal method was pre-specified in the Methods; reporting/publication and selection bias are addressed qualitatively in Section 4.2 (Study limitations).

Section and Topic	Item #	Checklist item	Location where item is reported
Certainty assessment	15	Describe any methods used to assess certainty (or confidence) in the body of evidence for an outcome.	No formal certainty rating (e.g., GRADE) was performed; limitations of the certainty of evidence are discussed narratively in Sections 4.1–4.2.
**RESULTS**			
Study selection	16a	Describe the results of the search and selection process, from the number of records identified in the search to the number of studies included in the review, ideally using a flow diagram.	Section 3.1 (Study selection): 774 records identified (PubMed 286; Scopus 239; Web of Science 249) → 27 studies included. Figure 1 (PRISMA flow diagram).
	16b	Cite studies that might appear to meet the inclusion criteria, but which were excluded, and explain why they were excluded.	Section 3.1 and Figure 1: full-text exclusions reported with reasons (unavailable data, n=9; failure to meet inclusion criteria, n=21). Excluded studies summarised by count/reason rather than individually cited.
Study characteristics	17	Cite each included study and present its characteristics.	Section 3.2 (Study Characteristics) and Table 1 (each included study cited with characteristics).
Risk of bias in studies	18	Present assessments of risk of bias for each included study.	Section 2.4 and Section 4.2: a formal per-study risk-of-bias assessment is not presented, given the predominance of case reports; the limitation is acknowledged.
Results of individual studies	19	For all outcomes, present, for each study: (a) summary statistics for each group (where appropriate) and (b) an effect estimate and its precision (e.g. confidence/credible interval), ideally using structured tables or plots.	Tables 1–4 present per-study/patient-level data (characteristics, procedural details, outcomes); Sections 3.2–3.5. Effect estimates with precision are not applicable (descriptive, non-comparative data).
Results of syntheses	20a	For each synthesis, briefly summarise the characteristics and risk of bias among contributing studies.	Sections 3.2–3.5 summarise contributing studies (predominantly case reports); risk-of-bias limitations noted in Section 4.2.
	20b	Present results of all statistical syntheses conducted. If meta-analysis was done, present for each the summary estimate and its precision (e.g. confidence/credible interval) and measures of statistical heterogeneity. If comparing groups, describe the direction of the effect.	Sections 3.3–3.5 (pooled descriptive results — e.g., technical success 32/39, 82.1%); Tables 2–4 and Figures 2–3. No meta-analytic summary estimates (descriptive synthesis only).
	20c	Present results of all investigations of possible causes of heterogeneity among study results.	Not applicable — no quantitative heterogeneity investigation (Section 2.5). Narrative comparison of IE vs. non-infected masses in Sections 3 and 4.
	20d	Present results of all sensitivity analyses conducted to assess the robustness of the synthesized results.	Not applicable — no sensitivity analyses performed.
Reporting biases	21	Present assessments of risk of bias due to missing results (arising from reporting biases) for each synthesis assessed.	Section 4.2 (Study limitations): publication and selection bias considered highly likely given the predominance of case reports; discussed narratively.
Certainty of evidence	22	Present assessments of certainty (or confidence) in the body of evidence for each outcome assessed.	No formal certainty (e.g., GRADE) rating presented; overall low/uncertain certainty is discussed in Sections 4.1–4.2.

Section and Topic	Item #	Checklist item	Location where item is reported
**DISCUSSION**			
Discussion	23a	Provide a general interpretation of the results in the context of other evidence.	Section 4.1 (Assessment of findings and additional literature).
	23b	Discuss any limitations of the evidence included in the review.	Section 4.2 (Study limitations): heterogeneous targets/techniques, inconsistent reporting, small samples.
	23c	Discuss any limitations of the review processes used.	Section 4.2: single-reviewer process, no formal meta-analysis, and pooling of biologically distinct lesions acknowledged.
	23d	Discuss implications of the results for practice, policy, and future research.	Section 4.1 (final paragraph — role as bridge/adjunct, call for prospective multicentre registries) and Section 5 (Conclusions).
**OTHER INFORMATION**			
Registration and protocol	24a	Provide registration information for the review, including register name and registration number, or state that the review was not registered.	Section 2.1: registered with the Open Science Framework (OSF), registration code 10.17605/OSF.IO/N384U [18].
	24b	Indicate where the review protocol can be accessed, or state that a protocol was not prepared.	Section 2.1: protocol registered/accessible via OSF (DOI 10.17605/OSF.IO/N384U), reference [18].
	24c	Describe and explain any amendments to information provided at registration or in the protocol.	No amendments to the registered protocol were made / none reported.
Support	25	Describe sources of financial or non-financial support for the review, and the role of the funders or sponsors in the review.	Funding statement: “not applicable” (no external funding received; no funder role).
Competing interests	26	Declare any competing interests of review authors.	Conflicts of Interest statement: the authors declare no conflict of interest.
Availability of data, code and other materials	27	Report which of the following are publicly available and where they can be found: template data collection forms; data extracted from included studies; data used for all analyses; analytic code; any other materials used in the review.	Data Availability Statement: “Not applicable.” All extracted study/patient-level data used in the synthesis are presented within the article (Tables 1–4). Protocol available via OSF (10.17605/OSF.IO/N384U).

From: Page MJ, McKenzie JE, Bossuyt PM, et al. The PRISMA 2020 statement: an updated guideline for reporting systematic reviews. BMJ 2021;372:n71. doi:10.1136/bmj.n71. For more information, visit http://www.prisma-statement.org/.
